# A Novel Scheme for DVL-Aided SINS In-Motion Alignment Using UKF Techniques

**DOI:** 10.3390/s130101046

**Published:** 2013-01-15

**Authors:** Wanli Li, Jinling Wang, Liangqing Lu, Wenqi Wu

**Affiliations:** 1 College of Mechatronics and Automation, National University of Defense Technology, Changsha 410073, China; E-Mails: luliangqing@hotmail.com (L.L.); wenqiwu_lit@hotmail.com (W.W.); 2 School of Surveying and Geospatial Engineering, University of New South Wales, Sydney, NSW 2052, Australia; E-Mail: jinling.wang@unsw.edu.au

**Keywords:** DVL-aided, in-motion alignment, AUKF, measurement noise covariance

## Abstract

In-motion alignment of Strapdown Inertial Navigation Systems (SINS) without any geodetic-frame observations is one of the toughest challenges for Autonomous Underwater Vehicles (AUV). This paper presents a novel scheme for Doppler Velocity Log (DVL) aided SINS alignment using Unscented Kalman Filter (UKF) which allows large initial misalignments. With the proposed mechanism, a nonlinear SINS error model is presented and the measurement model is derived under the assumption that large misalignments may exist. Since *a priori* knowledge of the measurement noise covariance is of great importance to robustness of the UKF, the covariance-matching methods widely used in the Adaptive KF (AKF) are extended for use in Adaptive UKF (AUKF). Experimental results show that the proposed DVL-aided alignment model is effective with any initial heading errors. The performances of the adaptive filtering methods are evaluated with regards to their parameter estimation stability. Furthermore, it is clearly shown that the measurement noise covariance can be estimated reliably by the adaptive UKF methods and hence improve the performance of the alignment.

## 1. Introduction

With the development of high-frequency, multi-beam Doppler sonar, which can provide bottom velocity measurements with a precision of 0.3% or less with a update rate of up to 5Hz, a wide variety of Doppler-based navigation techniques have been developed [1]. A typical navigation method for AUVs with minimal sonar use is based on a high quality SINS combined with occasional use of a Doppler Velocity Log [2]. This is an open-loop system so that the initial alignment is of great importance to subsequent navigation operation. In the case of an AUV, the initial alignment is more difficult. That is because the external aiding sensors such as GPS which provide geodetic-frame observations are unavailable for most of the time [3]. Therefore, it is an essential task to achieve an accurate alignment using DVL aiding within a very short period of time.

**Figure 1.**
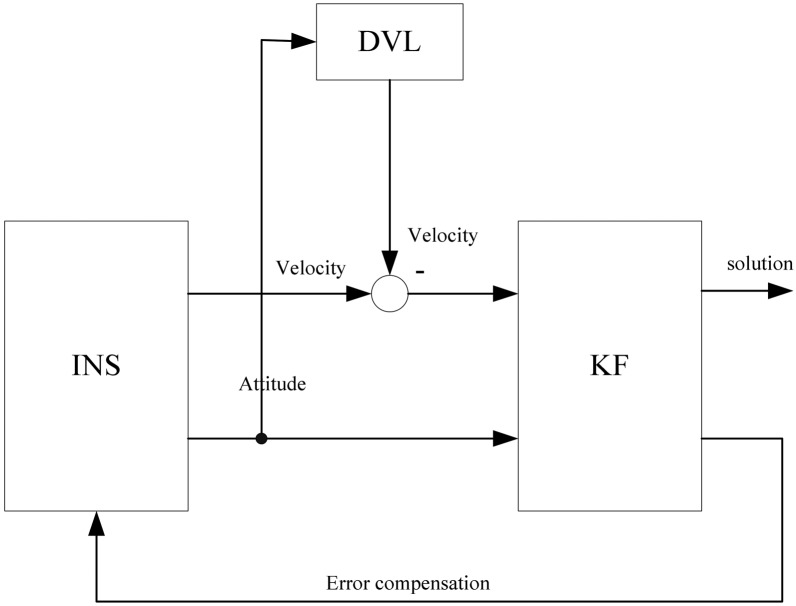
DVL-aided IMU alignment scheme.

Due to the random wave motions as well as the dynamics of the vehicle, it is difficult to estimate the attitude to the accuracy of within a few degrees in a short period with the existing coarse alignment methods [4]. Therefore, in-motion SINS alignment with large initial misalignments is always a challenge and thus needs to be considered. The difficulty for the DVL-aided alignment is that DVL provides the velocity measurements in the Doppler instrumental frame and hence could not be used as the measurement for alignment directly. There are mainly two alignment schemes to solve this problem for small misalignments [5,6].The first method is to establish the INS error dynamics in the body frame, so the velocity of the Doppler can be used as the measurements directly [5]. However, the INS error equations will include the unavoidable attitude error. Therefore, whether this method could be used for large misalignments problem needs to be analyzed. The other method is to establish the INS error dynamics in the navigation frame [6] and this scheme is shown in Figure 1. The velocity of Doppler is transformed to the navigation frame by the attitude matrix obtained from INS. Then it can be used as the measurements for the Kalman filter. The problem here is that the attitude errors are usually very large before the alignment is finished. It will cause a large measurement error that may lead to the divergence of the Kalman filter [5]. But the authors in [5] failed to notice that the measurement error could be compensated in the measurement model. By employing this scheme, a new alignment model which allows large misalignments is proposed in this study. From experimental results, the proposed alignment model is shown to be effective with any initial attitude.

The other main effort to deal with the large misalignments problem is based on such nonlinear filtering methods as the so-called extended Kalman filter (EKF), unscented Kalman filter (UKF), and particle filter (PF). Among these nonlinear filtering methods, the UKF is wildly used due to its elimination of the cumbersome derivation and low computational complexity [7]. The UKF is based on the unscented transformation (UT) which is founded on the intuition that an approximation of a probability distribution is easier than that of an arbitrary nonlinear function [8]. It is able to capture the true mean and covariance of the Gaussian Random Variable to the 3rd order accuracy [9] and hence proved to be superior to EKF [10]. The most obvious shortcoming of EKF is that it requires the computation of Jocobian matrices and linear approximations of the EKF can be very inaccurate in some scenarios, leading to filter instability [9,11]. Similar to the KF [12–14] and EKF, covariance parameters also play an important role in the filtering performance. Therefore, several adaptive filtering techniques are developed to improve the performance of the UKF [15]. The covariance-matching method which is based on the concept of making the elements of the actual innovation covariance matrix consistent with their theoretical values has been shown to be one of the most promising techniques for KF [16]. This method is extended to UKF for its application in nonlinear systems [17]. However, it could not be guaranteed that the resulting measurement noise covariance is positive definite. Inspired by the work in [18], another covariance-matching adaptive filtering method based on the filter residual sequence is presented. This study first evaluates the impacts of adaptive filtering methods on the parameter estimation stability with different window sizes and different initial measurement noise covariance matrix. Experimental results demonstrate that the measurement noise covariance can be estimated reliably by the adaptive filtering methods. The performance of the adaptive filtering methods are also compared and analyzed.

The rest of this paper is organized as follows: Section 2 is devoted to the presentation of the nonlinear DVL-aided IMU alignment model which can tolerate large misalignments. Section 3 presents the mathematical formulas of the UKF and adaptive UKF techniques. In Section 4, the performance of the proposed algorithms are evaluated and compared with real experimental data. The conclusions are drawn in Section 5.

## 2. Alignment Model

### 2.1. INS Error Dynamics Model

The nonlinear SINS error model proposed in [19,20] will be used in this paper. The nominal error state includes the velocity error (*δv^c^*), the attitude error (*ψ*), the accelerometer bias (∇*^b^*) and the gyro bias (*ε^b^*). The formulas of this model are given as follows:

(1) $$\delta {\dot{v}}^{c}=(I-C_{p}^{c})C_{b}^{p}f^{b}-(2\Omega_{ie}^{c}+\Omega_{ec}^{c})\delta v^{c}-\delta \Omega_{ec}^{c}v^{c}+{\text{C}}_{b}^{c}\nabla^{b}+\delta g^{c}$$

(2) $$\dot{\psi }=(I-{\text{C}}_{c}^{p})\omega_{ic}^{c}-{\text{C}}_{b}^{p}\varepsilon^{b}$$

(3) $${\dot{\nabla }}^{b}=0$$

(4) $${\dot{\varepsilon }}^{b}=0$$

where the superscript *c* denotes the local level frame at the computed position and its orientation is north-up-east (NUE). *p*, *i* and *b* are the platform frame, the inertial frame and the body frame respectively. *C* is the direction cosine matrix. *f ^b^* is the accelerometer measurement. *δg^c^* is the gravity error caused by the position error and it can be ignored during the process of the alignment. $\Omega_{ie}^{c}$ is the skew matrix of $\omega_{ie}^{c}$ which is the angle rate of the Earth frame relative to the inertial frame. $\omega_{ie}^{c}$ is given by:

(5) $$\omega_{ie}^{c}=\left[\begin{array}{c} \omega_{ie}\cos l\\ \omega_{ie}\sin l\\ 0 \end{array}\right]$$

where: *l* is the geographic latitude; *ω_ie_* is the earth's rotation angular velocity. $\Omega_{ec}^{c}$ is the skew matrix of $\omega_{ec}^{c}$ which is the angle rate of the navigation frame relative to the Earth frame. $\omega_{ec}^{c}$ is given by:

(6) $$\omega_{ec}^{c}=\left[\begin{array}{c} \frac{V_{E}}{R_{n}}\\ \frac{V_{E}\tan l}{R_{n}}\\ -\frac{V_{N}}{R_{m}} \end{array}\right]$$

where *R_n_* and *R_m_* are the transverse and meridian radius of the curvature respectively; the subscripts *E* and *N* denote the east and north components in the *c* frame. $\omega_{ic}^{c}$ is the angle rate of the navigation frame relative to the inertial frame. It can be obtained by:

(7) $$\omega_{ic}^{c}=\omega_{ie}^{c}+\omega_{ec}^{c}$$

Define:

(8) $$\left[\begin{array}{c} s_{x}\\ c_{x}\\ s_{y}\\ c_{y}\\ s_{z}\\ c_{z} \end{array}\right]=\left[\begin{array}{c} \sin(\psi_{x})\\ \cos(\psi_{x})\\ \sin(\psi_{y})\\ \cos(\psi_{y})\\ \sin(\psi_{z})\\ \cos(\psi_{z}) \end{array}\right]$$

${\text{C}}_{p}^{c}$ is given by:

(9) $${\text{C}}_{p}^{c}=\left[\begin{array}{ccc} c_{z}c_{y} & s_{x}s_{y}-c_{x}s_{z}c_{y} & c_{x}s_{y}+s_{x}s_{z}c_{y}\\ s_{z} & c_{x}c_{z} & -s_{x}c_{z}\\ -c_{z}s_{y} & s_{x}c_{y}+c_{x}s_{z}s_{y} & c_{x}c_{y}-s_{x}s_{z}s_{y} \end{array}\right]$$

In the error model presented above, all the three misalignment angles are assumed to be large. For real time applications, it is often the case that there are a large uncertainty in heading angle and low uncertainties in leveling angles [20].

### 2.2. Measurement Model

The velocity of Doppler in the local level frame ${\text{v}}_{d}^{c}$ can be described as follows:

(10) $${\text{v}}_{d}^{c}={\text{C}}_{b}^{c}{\text{C}}_{d}^{b}v_{d}$$

where ${\text{C}}_{d}^{b}$ is a constant matrix which translates the velocity of Doppler *v_d_* from the Doppler instrumental frame to the vehicle body frame. It needs to be calibrated before a mission is conducted [21]. Supposing the error of ${\text{v}}_{d}^{c}$ is mainly caused by the platform misalignments, the DVL measurement in error ${\overset{⋜}{v}}_{d}^{c}$ can be described as follows:

(11) $$\begin{array}{c} {\tilde{v}}_{d}^{c}={\text{C}}_{b}^{c}{\text{C}}_{d}^{b}v_{d}+\delta {\text{C}}_{b}^{c}{\text{C}}_{d}^{b}v_{d}\\ ={\text{v}}_{d}^{c}+\delta {\text{C}}_{b}^{c}{\text{C}}_{d}^{b}v_{d} \end{array}$$

where the perturbation of the attitude matrix $\delta {\text{C}}_{b}^{c}$ is given by [22]:

(12) $$\delta {\text{C}}_{b}^{c}=(I-{\text{C}}_{c}^{p}){\text{C}}_{b}^{c}$$

Inserting Equation (12) into Equation (11), it yields:

(13) $${\tilde{v}}_{d}^{c}={\text{v}}_{d}^{c}+(I-{\text{C}}_{c}^{p}){\text{C}}_{b}^{c}{\text{C}}_{d}^{b}v_{d}$$

Differentiating the velocity of INS and DVL, the measurement model is given below:

(14) $$\begin{array}{ll} {\tilde{v}}_{\text{INS}}^{c}-{\tilde{v}}_{d}^{c} & =v_{\text{INS}}^{c}+\delta v_{\text{INS}}^{c}-{\text{v}}_{d}^{c}-(I-{\text{C}}_{c}^{p}){\text{C}}_{b}^{c}{\text{C}}_{d}^{b}v_{d}\\ & =\delta v_{\text{INS}}^{n}-(I-{\text{C}}_{c}^{p}){\text{C}}_{b}^{c}{\text{C}}_{d}^{b}v_{d} \end{array}$$

## 3. UKF Techniques

### 3.1. UKF in Additive Noise Case

The considered nonlinear discrete-time system with additive noise is presented as follows [23]:

(15) $$x_{k}=f(x_{k-1})+w_{k-1}$$

(16) $$z_{k}=h(x_{k})+\upsilon_{k}$$

where *x_k_* ∊ *R^n^* is the state vector; *z_k_* ∊ *R^m^* is the measurement vector; *f*(·) and *h*(·) are nonlinear functions; *w_k_* and *υ_k_* are the uncorrelated zero-mean Gaussian white sequences and their conversances are:

(17) $$\begin{array}{l} E[w_{k}{\text{w}}_{j}^{T}]=\delta_{kj}Q_{k}\\ E[\upsilon_{k}\upsilon_{j}^{T}]=\delta_{kj}R_{k}\\ E[w_{k}\upsilon_{j}^{T}]=0 \end{array}$$

With a view of reducing the computational burden, the non-augmented UKF is widely used in such additive noise cases [7,24–26]. The resemblance between the UKF and the KF is that the implementations of the two algorithms all consist of the prediction of the state mean and covariance, and then the update with the measurements [27]. The implementation of the non-augmented UKF algorithm is given as follows [25,26]:

- Initialization:
  (18) $${\hat{x}}_{0}=E[x_{0}],P_{0}=E\left[({\hat{x}}_{0}-x_{0}){\left({\hat{x}}_{0}-x_{0}\right)}^{T}\right]$$
- Time-updating:
  (19) $$\chi_{k-1}=\left[\begin{array}{ccc} {\hat{x}}_{k-1} & {\left[{\hat{x}}_{k-1}\right]}_{L}+\gamma \sqrt{P_{k-1}} & {\left[{\hat{x}}_{k-1}\right]}_{L}-\gamma \sqrt{P_{k-1}} \end{array}\right]$$
  (20) $$\chi_{i,k\;|\;k-1}^{*}=f(\chi_{i,k-1})$$
  (21) $${\hat{x}}_{k\;|\;k-1}=\sum_{i=0}^{2L}W_{i}^{\left(m\right)}\chi_{i,k\;|\;k-1}^{*}$$
  (22) $$P_{k\;|\;k-1}=\sum_{i=0}^{2L}W_{i}^{\left(c\right)}\left(\chi_{i,k\;|\;k-1}^{*}-{\hat{x}}_{k\;|\;k-1}\right){\left(\chi_{i,k\;|\;k-1}^{*}-{\hat{x}}_{k\;|\;k-1}\right)}^{T}+Q_{k-1}$$
  (23) $$\chi_{k\;|\;k-1}=\left[\begin{array}{ccc} {\hat{x}}_{k\;|\;k-1} & {\left[{\hat{x}}_{k\;|\;k-1}\right]}_{L}+\gamma \sqrt{P_{k\;|\;k-1}} & {\left[{\hat{x}}_{k\;|\;k-1}\right]}_{L}-\gamma \sqrt{P_{k\;|\;k-1}} \end{array}\right]$$
  (24) $$\eta_{i,k\;|\;k-1}=h(\chi_{i,k\;|\;k-1})$$
  (25) $${\hat{z}}_{k\;|\;k-1}=\sum_{i=0}^{2L}W_{i}^{\left(m\right)}\eta_{i,k\;|\;k-1}$$
- Measurement-updating:
  (26) $$P_{{\hat{z}}_{k}{\hat{z}}_{k}}=\sum_{i=0}^{2L}W_{i}^{\left(c\right)}\left(\eta_{i,k\;|\;k-1}-{\hat{z}}_{k\;|\;k-1}\right){\left(\eta_{i,k\;|\;k-1}-{\hat{z}}_{k\;|\;k-1}\right)}^{T}+R_{k}$$
  (27) $$P_{{\hat{x}}_{k}{\hat{z}}_{k}}=\sum_{i=0}^{2L}W_{i}^{\left(c\right)}\left(\chi_{i,k\;|\;k-1}-{\hat{x}}_{k\;|\;k-1}\right){\left(\eta_{i,k\;|\;k-1}-{\hat{z}}_{k\;|\;k-1}\right)}^{T}$$
  (28) $$K_{k}=P_{{\hat{x}}_{k}{\hat{z}}_{k}}P_{{\hat{z}}_{k}{\hat{z}}_{k}}^{-1}$$
  (29) $${\hat{x}}_{k}={\hat{x}}_{k\;|\;k-1}+K_{k}\left(z_{k}-{\hat{z}}_{k\;|\;k-1}\right)$$
  (30) $$P_{k}=P_{k\;|\;k-1}-K_{k}P_{{\hat{z}}_{k}{\hat{z}}_{k}}{\text{K}}_{k}^{T}$$

The parameters for calculating the sigma-points are given as follows:

(31) $$\{\begin{array}{c} \lambda =\alpha^{2}\left(L+\kappa \right)-L\\ \gamma =\sqrt{L+\lambda }\\ W_{0}^{\left(m\right)}=\lambda /\left(L+\lambda \right)\\ W_{0}^{\left(c\right)}=\lambda /\left(L+\lambda \right)+\left(1-\alpha^{2}+\beta \right)\\ W_{i}^{\left(m\right)}=W_{i}^{\left(c\right)}=1/\left[2\left(L+\lambda \right)\right],\left(i=1,2,\ldots ,2L\right) \end{array}$$

where *W*^(^*^m^*^)^ and *W*^(^*^c^*^)^ represent the mean weight and covariance weight, respectively [15]; *L* is the dimension of the state vector; $\sqrt{P_{k\;|\;k-1}}$ is the *i* th column of the matrix square root of *P_k_*_∣_*_k_*_−1_; *α* determines the spread of the sigma points around *x̄* and is usually set to a small positive value (e.g., 1e-3); *κ* is a secondary scaling parameter which is usually set to 0; *β* is used to incorporate prior knowledge of the distribution of *x* (for Gaussian distributions, *β* = 2 is optimal) [28].

### 3.2. Innovation-Based Adaptive UKF

The innovation sequence *d_k_* which is the difference between the measurement *z_k_* and its predicted value *ẑ_k_*_∣_*_k_*_−1_ is given as follows:

(32) $$d_{k}=z_{k}-{\hat{z}}_{k\;|\;k-1}$$

where *ẑ_k_*_∣_*_k_*_−1_ is obtained from Equation (25). By matching the covariance matrix of the innovation sequence with its theoretical form, *P_ẑ_k_ẑ_k__* can be calculated as follows [17]:

(33) $$P_{{\hat{z}}_{k}{\hat{z}}_{k}}=\frac{1}{N}\sum_{j=k-N+1}^{k}d_{j}{\text{d}}_{j}^{T}$$

*N* is the length of the sliding sampling window. By replacing the Equation (26) with the Equation (33), the Adaptive UKF (AUKF) algorithm is obtained. This technique is similar to the traditional innovation-based adaptive Kalman filter (AKF) [16,29]. By comparing the Equation (33) with (26), the estimation of the measurement noise covariance can be obtained as follows:

(34) $$R_{k}=\frac{1}{N}\sum_{j=k-N+1}^{k}d_{j}{\text{d}}_{j}^{T}-\sum_{i=0}^{2L}W_{i}^{\left(c\right)}\left(\eta_{i,k\;|\;k-1}-{\hat{z}}_{k\;|\;k-1}\right){\left(\eta_{i,k\;|\;k-1}-{\hat{z}}_{k\;|\;k-1}\right)}^{T}$$

As can be seen from Equation (34), the estimated measurement noise covariance is the difference between two positive definite matrices, it cannot be guaranteed that the resulting matrix *R_k_* is positive definite. This trouble can easily cause the failure of the filter in real time applications.

### 3.3. Residual-Based Adaptive UKF

The residual sequence could also be used with the purpose of obtaining a realistic estimator of the measurement noise covariance. The residual sequence *ε_k_* is defined as follows:

(35) $$\varepsilon_{k}=z_{k}-{\hat{z}}_{k}$$

where *Ẑ_k_* can be obtained by the estimated values *X̂_k_* (not the predicted values *X̂_k_*_|_*_k_*_−1_) by an extra UT:

(36) $$\chi_{k}=\left[\begin{array}{ccc} {\hat{x}}_{k} & {\left[{\hat{x}}_{k}\right]}_{L}+\gamma \sqrt{P_{k}} & {\left[{\hat{x}}_{k}\right]}_{L}-\gamma \sqrt{P_{k}} \end{array}\right]$$

(37) $$\eta_{i,k}=h(\chi_{i,k})$$

(38) $${\hat{z}}_{k}=\sum_{i=0}^{2L}W_{i}^{\left(m\right)}\eta_{i,k}$$

by extending concept of the residual-based AKF [18] to UKF, a recursive estimator of *R_k_* can be obtained as:

(39) $$R_{k}=\frac{1}{N}\sum_{j=k-N+1}^{k}\varepsilon_{j}\varepsilon_{j}^{T}+\sum_{i=0}^{2L}W_{i}^{\left(c\right)}\left(\eta_{i,k}-{\hat{z}}_{k}\right){\left(\eta_{i,k}-{\hat{z}}_{k}\right)}^{T}$$

It can be used in the computation of epoch *k* + 1. Compared to Equation (34), as Equation (39) is the sum of two positive definite matrices, the estimated covariance matrix is always positive definite. A slight disadvantage of this method is that it requires some extra computation for *ẑ_k_* which is not generated by the standard UKF process. The residual-based method utilizes the measurement of the previous *N* epoch, whereas the innovation-based method utilizes current and the previous *N*-1 epoch. Therefore, the innovation sequence and the residual sequence have to be ergodic and stationary over the *N* steps. Otherwise, the performance of UKF will be degraded.

## 4. Experimental Results and Discussions

### 4.1. Test Configuration

The ship-mounted experimental data were collected to evaluate the performance of the in-motion alignment. The experiment was carried out in Yangzi River. The equipped sensors are listed as follows:

- IMU: Consists of three ring laser gyroscopes with drift rate 0.01° / *h*(1*σ*) and three quartz accelerometers with bias 5×10^−5^*g*(1*σ*). Its update rate is 200 Hz.
- Bottom-lock Doppler: Provides three-axis transformation velocities with accuracy ±5‰ of speed and update rates up to 1 Hz.
- GPS receiver: Provides velocity with precision of about 0.1m/s, position with precision of about 10 m, and update rates up to 1 Hz.

**Figure 2.**
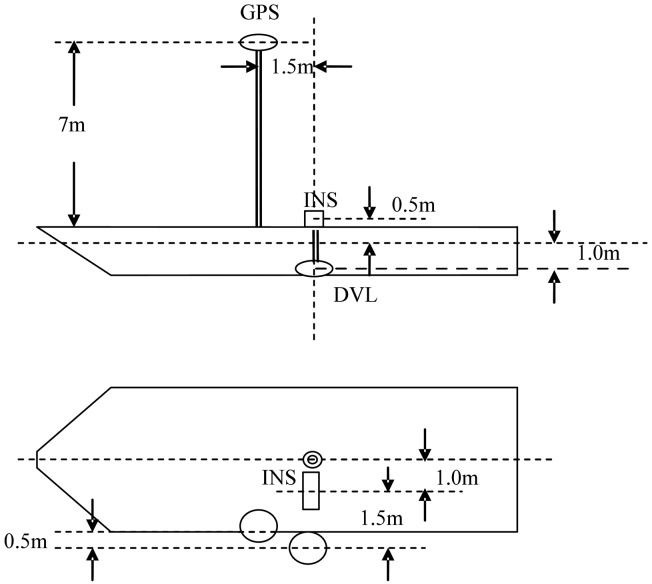
Fixing of experimental devices.

**Figure 3.**
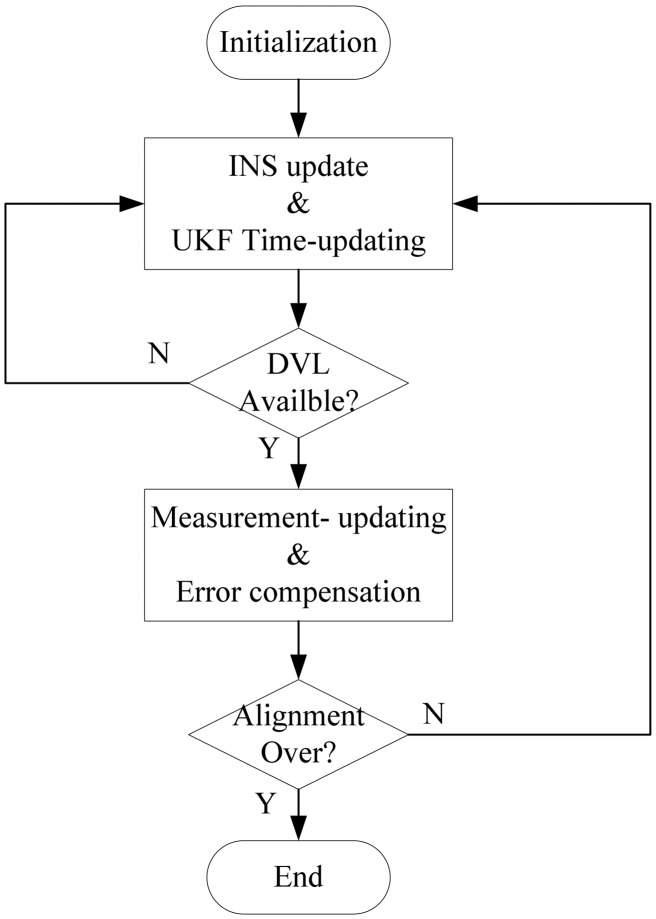
Alignment flowchart.

In the experiment, the IMU and the GPS receiver were set up on a vessel. The DVL module was put beneath 1m underwater. The fixing and level arm parameters of devices are shown in Figure 2. The flowchart of the alignment procedure is shown in Figure 3. As the update rate of the INS is much higher than DVL, the measurement-updating is executed only when the DVL is available. By using the close-loop filtering scheme, the filtering corrections are fed back every measurement-updating. If the filtering epoch is smaller than the sampling window size of innovation or residual, AUKF is working at the mode of UKF.

### 4.2. Alignment Results by UKF

**Figure 4.**
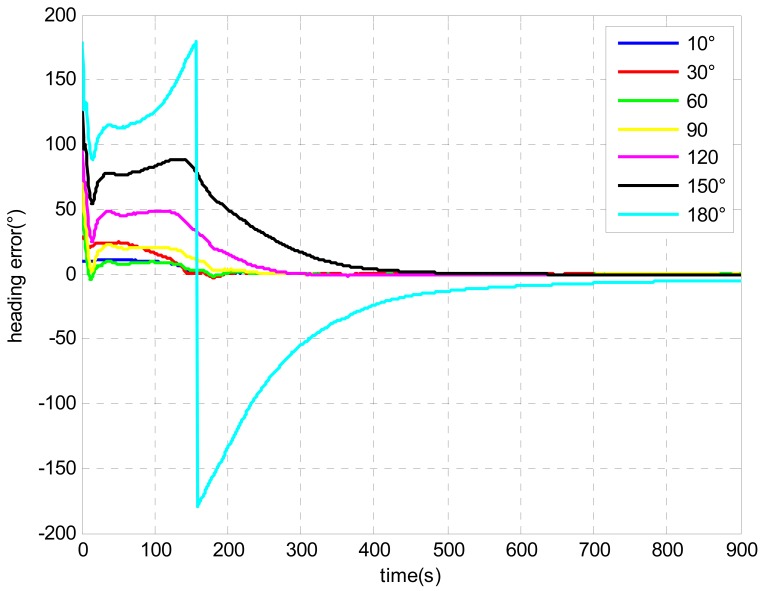
Heading error comparison with different initial heading errors.

Figure 4 shows the error curves of heading with initial heading errors from 10 degrees to 180 degrees. As can be seen from the figure, all the error curves converged with time. The proposed alignment model is shown to be effective with any initial attitude.

**Figure 5.**
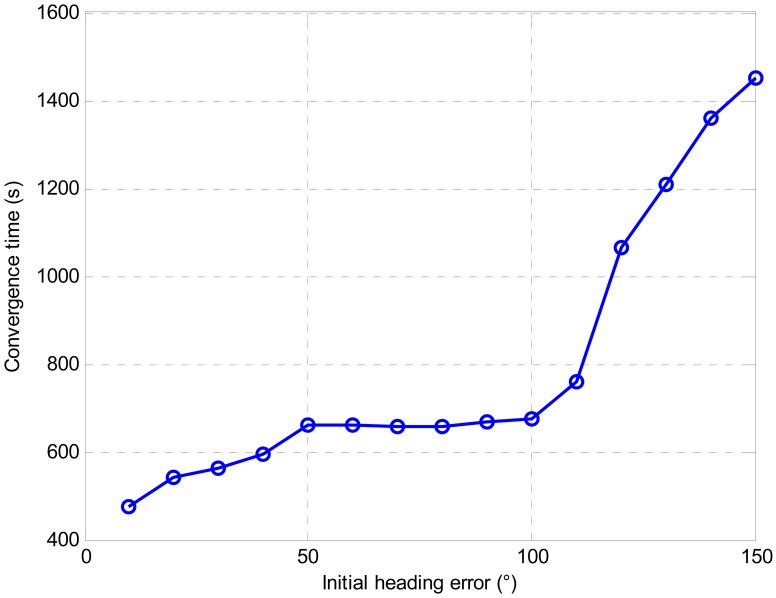
Convergence time comparison with different initial heading errors.

**Figure 6.**
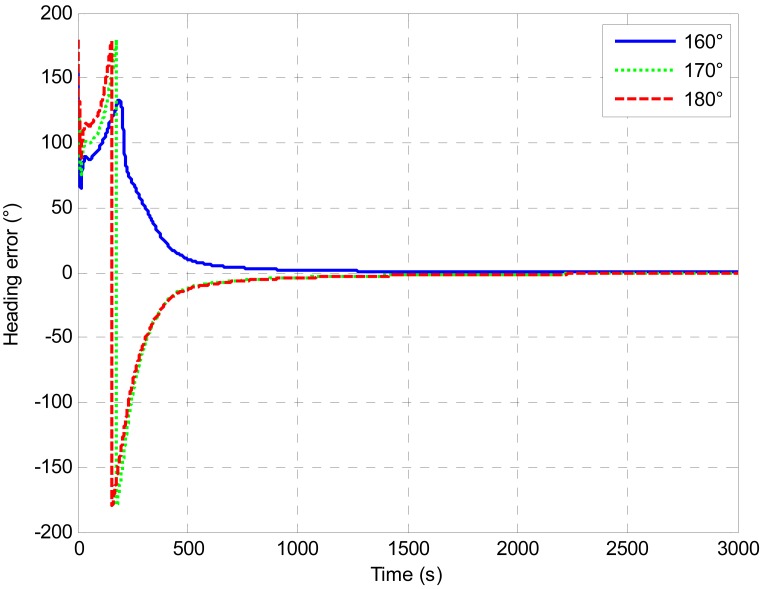
Heading error comparison with extra large initial heading errors.

Figure 5 shows convergence time for different initial heading errors with a converged heading error of less than 0.1 degree. It is clearly shown that larger initial heading error needs more time to converge. In addition, if the initial heading error is larger than 110 degrees. It is hard to guarantee that the final converged heading error would reach 0.1 degree within the 900 s alignment. Figure 6 shows the heading error comparison with extra large initial heading errors. The alignment time was extended to 3,000 s. Though all the estimates converged with time, the heading errors were 0.23 degree, 0.72 degree and 0.73 degree with initial heading error of 160 degrees, 170 degrees and 180 degrees respectively. It is clearly shown that the heading errors converged faster at the beginning and then the speed of the convergence slowed down. This was caused by the fading of the Kalman Filter gain [30]. Therefore, further study is still needed to improve the performance of the UKF. Moreover, providing a relatively accurate initial attitude will be beneficial to the alignment.

### 4.3. Measurement Noise Covariance Estimation

**Figure 7.**
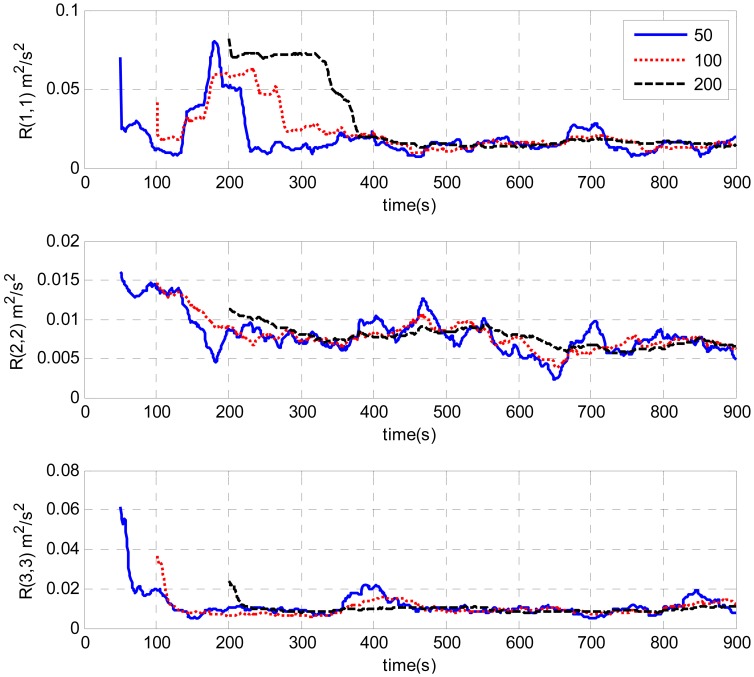
Estimation of measurement noise covariance with different window sizes by innovation-based AUKF.

**Figure 8.**
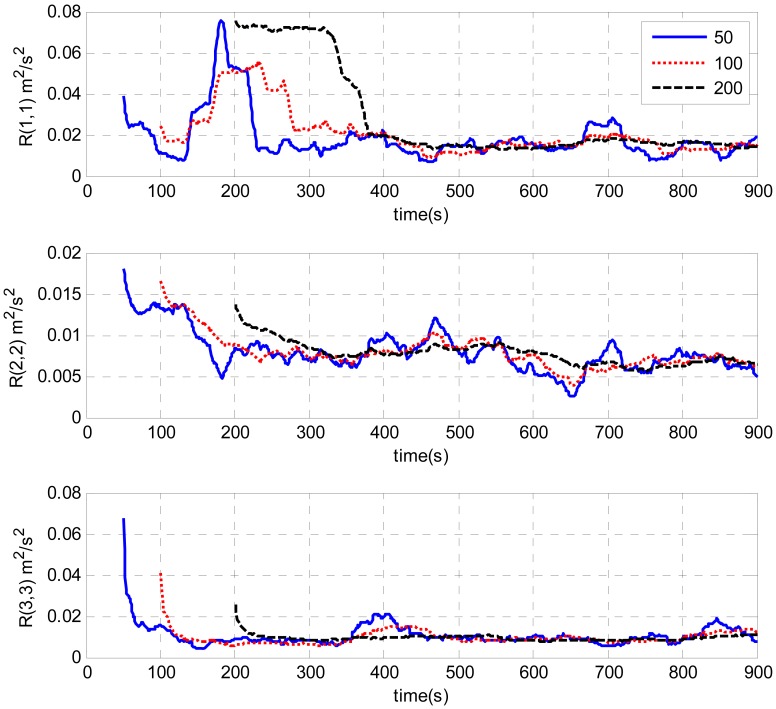
Estimation of measurement noise covariance with different window sizes by residual-based AUKF.

A test is done by intentionally adding large initial attitude errors (100 degrees for heading, 1 degree for roll and pitch). Figures 7 and 8 show the estimations of measurement noise covariance with different window sizes by the innovation-based and the residual-based AUKF respectively. The trends of the estimations obtained by these two methods are similar. As can be seen from the figures, the estimations of the measurement noise are unstable at the beginning and then converge with time. Finally, all the estimates converge to the value of around 0.01 (m^2^/s^2^). It can also be seen from the figures that the estimation oscillation becomes obvious when shorter window sizes are used. It illustrates that a short window size may lead to unstable estimation. This is similar to the conclusions of AKF [16].

A test is done by intentionally adding large initial attitude errors (100 degrees for heading, 1 degree for roll and pitch). Figures 7 and 8 show the estimations of measurement noise covariance with different window sizes by the innovation-based and the residual-based AUKF respectively. The trends of the estimations obtained by these two methods are similar. As can be seen from the figures, the estimations of the measurement noise are unstable at the beginning and then converge with time. Finally, all the estimates converge to the value of around 0.01 (m^2^/s^2^). It can also be seen from the figures that the estimation oscillation becomes obvious when shorter window sizes are used. It illustrates that a short window size may lead to unstable estimation. This is similar to the conclusions of AKF [16].

**Figure 9.**
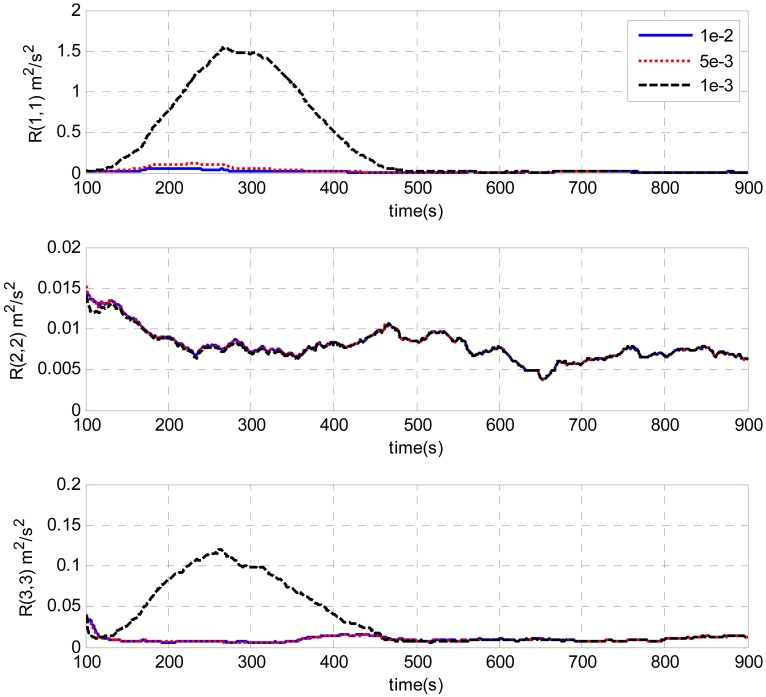
Estimation of measurement noise covariance with different initial *R* values by innovation-based AUKF.

**Figure 10.**
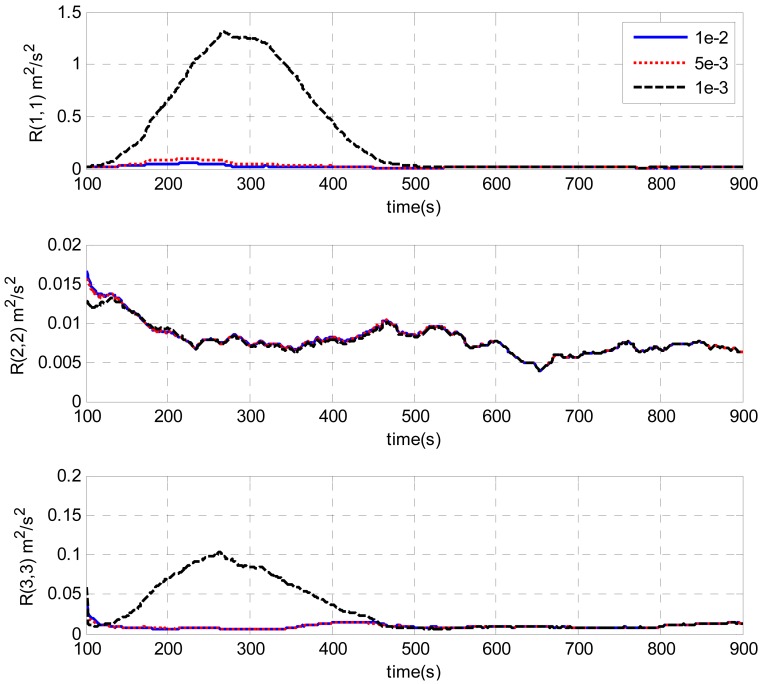
Estimation of measurement noise covariance with different initial *R* values by residual-based AUKF.

Figures 9 and 10 illustrate the estimates of the measurement noise covariance with different initial *R* by the AUKF techniques. The window size used for estimation was 100. It is clearly shown that the initial values of *R* have some impacts only during the filter convergence periods. Finally, all the curves converged and closely matched each other. The requirement of a priori knowledge of the measurement noise covariance is alleviated. In addition, it is clearly shown by Figures 7–10, the estimated measurement noise covariance obtained by the innovation based method almost approaches that obtained by the residual based case. There is no decided difference between them.

### 4.4. Performance Evaluation of the Adaptive UKF Techiniques

**Figure 11.**
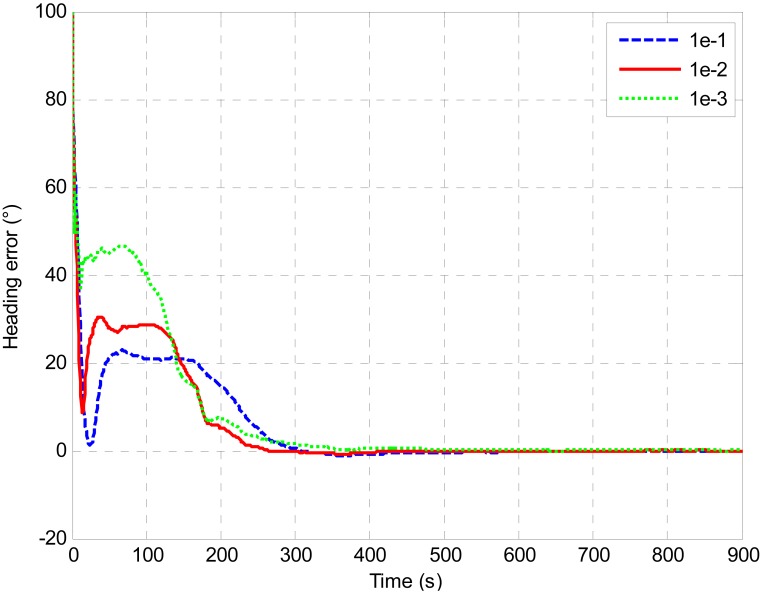
Heading error comparisons with different *R*.

A test was designed to evaluate the performance of the estimated measurement noise covariance. The initial attitude error was 100 degrees for heading, 1 degree for roll and pitch, respectively. As can be seen from Figures 7 and 8, the values of the estimated measurement noise covariance were around 0.01 (m^2^/s^2^). Therefore, this value was applied in the alignment [*R* = diag(0.01,0.01,0.01) (m^2^/s^2^)]. In addition, the alignment was also executed with a larger *R* [*R* = diag(0.1,0.1,0.1) (m^2^/s^2^)] and a smaller *R* [*R* = diag(0.001,0.001,0.001) (m^2^/s^2^)]. Figure 11 shows the error curves of the heading with different *R*. As can be seen from the figure, all the estimates converged with time. But the heading error with *R* value of 0.01 (m^2^/s^2^) converged more rapidly than that with the value of 0.1 (m^2^/s^2^) and 0.001 (m^2^/s^2^).As shown in Table 1, it took 676 seconds for the heading error to converge within 0.1 degree with *R* value of 0.01 (m^2^/s^2^) while the convergence time for *R* value of 0.1 (m^2^/s^2^) and 0.001 (m^2^/s^2^) were 766 s and 800 s respectively. In a sense, the estimated measurement noise covariance is proved to be realistic. Furthermore, it's clearly shown that the measurement noise covariance plays an important part in the performance of the UKF. Once the *R* is correctly estimated, it can improve the performance of the alignment.

**Figure 12.**
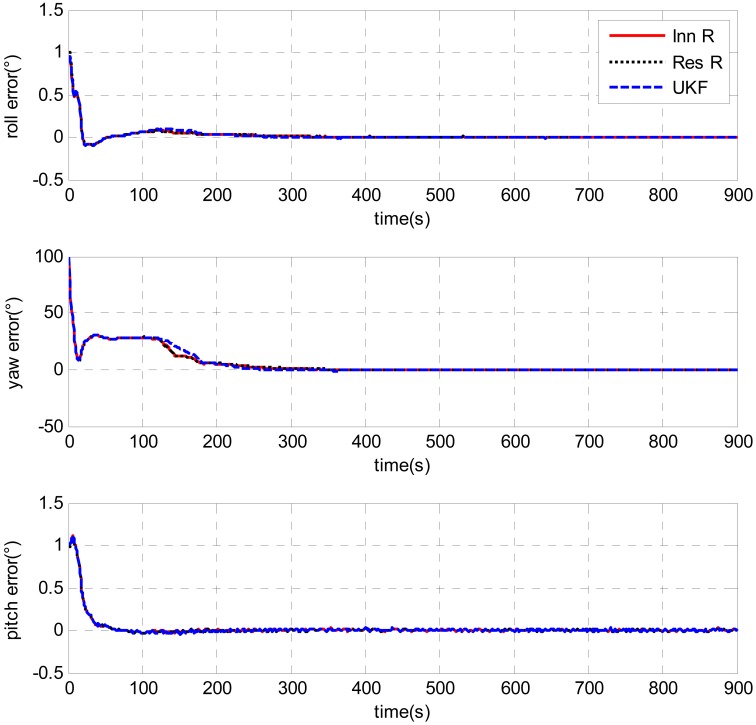
Attitude error comparison between UKF and AUKFs with initial attitude error of [1°, 100°, 1°].

**Figure 13.**
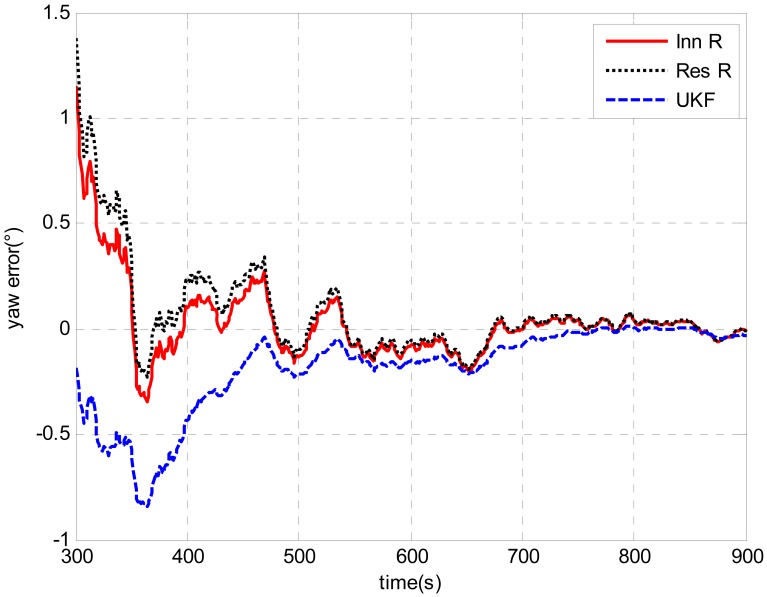
Partial magnification of the heading error.

The tests were designed to compare the performance of the UKF and AUKF techniques for their applications in the DVL-aided SINS alignment problem. An example is shown in Figure 12. In the test, the initial attitude error was 100 degrees for heading and 1 degree for leveling. The initial *R* value applied in UKF and AUKF was 0.01 (m^2^/s^2^). The window size for both the innovation-based and residual-based AUKF was 100. As can be seen from Figure 12, the estimated results of the UKF and the AUKF were different after 100 s when the AUKF methods were switched to the adaptive algorithms. Due to the setting of *R* [0.01 (m^2^/s^2^)] given for the UKF was very close to the optimal values obtained from the above tests, the trends of the error curves obtained by the UKF and the AUKF methods are similar. Partial magnification of the heading errors are shown in Figure 13. It is clearly shown that the heading error estimated by the UKF closely matches that predicted by the AUKF methods with the increase of the time. It means that the distinctions between the performances of the UKF and the AUKF are small if a appropriate measurement noise covariance [*R* = diag(0.01,0.01,0.01) (m^2^/s^2^)] is applied. However, in the case of underwater vehicles, it's very difficult to obtain a prior knowledge of the measurement noise covariance due to unstable Doppler measurements. Though a proper *R* is employed, AUKF methods still performed a little better UKF. Compared with 676 seconds, it took 667 and 664 seconds for the heading errors to converge within 0.1 degree by the innovation based and the residual based AUKF, respectively. As can be seen from Figure 13, their final heading errors are also smaller than UKF. In addition, there is no notable contrast between the AUKF methods. The error curve that predicted by innovation based AUKF is exactly parallel to that obtained by residual based AUKF.

**Figure 14.**
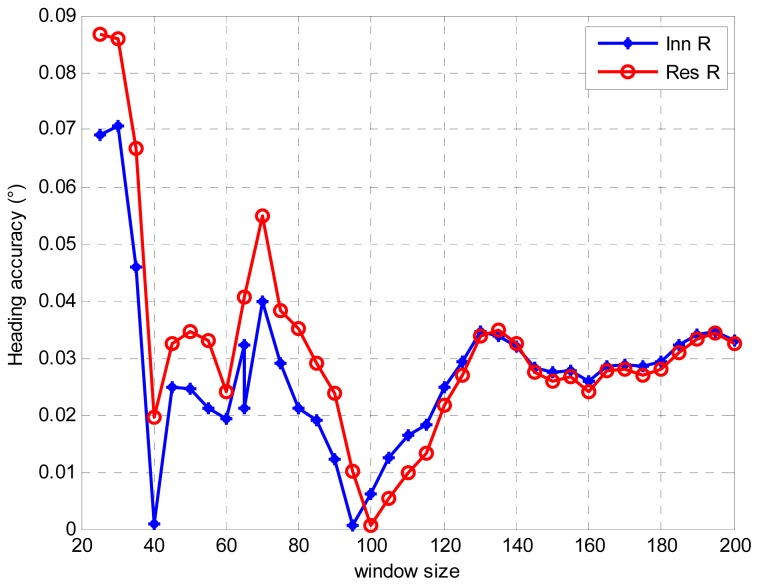
Heading accuracy comparison with different window sizes.

Regarding the attitude obtained from a high precision INS/GPS integration as reference, Figure 14 shows the heading accuracy comparison with different window sizes in the 900 s alignments. It is clearly shown that the estimated results meet the requirement of the alignment of 0.1 degree for the heading error. Choosing an optimal window size is of great importance. However, how to determine the optimal window size is still a challenge issue. It can now only be determined by simulations or experiences. As can be seen from Figure 14, the trends of the curves obtained by the innovation-based and residual-based AUKFs are similar. Accompanying with oscillations, the heading accuracy slowly increased until the window size was around 100. After that, the heading accuracy slowly decreased to 0.03 degree with larger the window size. Then, the heading accuracy remained around 0.03 degree. In addition, the oscillations was obvious when the window size was smaller than 100. This was caused by the unstable estimation of the measurement noise covariance with small window sizes. The result presented in Figure 14 meets our knowledge that the small window size will lead to the biased estimation of the measurement noise covariance while very large window size may cause the adaptive filter loosing the ability of adaptation [29]. Moreover, for this specific case, a slight difference between the innovation-based and residual-based AUKF methods is that the window size of 95 performed best for innovation-based AUKF while window size of 100 performed best for the residual-based AUKF.

## 5. Conclusions

This paper has presented a new alignment scheme for the DVL-aided SINS in-motion alignment which allows large initial misalignments. From the experimental data, it has been clearly shown that the proposed alignment model can be applied for the DVL-aided SINS in-motion alignment with any initial heading errors. As the measurement noise covariance is of great importance to the performance of the UKF, the covariance-matching methods applied in AKF have been extended for use in the Adaptive UKF. By using innovation-based and residual-based AUKF techniques, the measurement noise covariance can be estimated reliably and hence can improve the performance of the UKF. Its performance has been demonstrated with field experimental data.

## Figures and Tables

**Table 1.** Performance comparisons with different *R* values.

**R Value (m^2^/s^2^)**	**Heading Accuracy (°)**	**Convergence Time (s)**
1e-1	0.0629	766
1e-2	0.0282	676
1e-3	0.0367	800
